# The prevalence and social patterning of chronic diseases among older people in a population undergoing health transition. A 10/66 Group cross-sectional population-based survey in the Dominican Republic

**DOI:** 10.1186/1471-2458-10-344

**Published:** 2010-06-16

**Authors:** Daisy Acosta, Ruth Rottbeck, Juana G Rodríguez, Loida M González, Mary R Almánzar, Susana N Minaya, Maria del C Ortiz, Cleusa P Ferri, Martin J Prince

**Affiliations:** 1 Internal Medicine Department, Geriatric Section, Universidad Nacional Pedro Henriquez Ureña (UNPHU), John F Kennedy Avenue, Santo Domingo, Dominican Republic; 2 Centre Hospitalier, Universitaire de Butare, Butare, Rwanda; 3 Ministerio De Salud Pública y Asistencia Social Dirección Área VI De Salud, Calle #28, esquina #39, Ensanche La Fé, Santo Domingo, Dominican Republic; 4 Laboratorio Nacional Dr Defilló, Calle Santo Thomás de Aquino #1, Santo Domingo, Dominican Republic; 5 Health Service and Population Research Department, Institute of Psychiatry P060, De Crespigny Park, London SE5 8AF, UK

## Abstract

**Background:**

Very little of the increased attention towards chronic diseases in countries with low and middle incomes has been directed towards older people, who contribute 72% of all deaths, and 14% of all Disability Adjusted Life Years linked to this group of conditions in those regions. We aimed to study the prevalence of physical, mental and cognitive diseases and impairments among older people in the Dominican Republic, their social patterning, and their relative contributions to disability.

**Methods:**

A cross-sectional catchment area one-phase survey of chronic disease diagnoses, physical impairments, risk factors and associated disability among 2011 people aged 65 years and over (of whom 1451 gave fasting blood samples) in Santo Domingo, Dominican Republic.

**Results:**

The most prevalent diagnoses were hypertension (73.0%), anaemia (35.0%), diabetes (17.5%), depression (13.8%) and dementia (11.7%), with 39.6% meeting criteria for metabolic syndrome. After direct standardization (for age and sex) the prevalences of stroke (standardized morbidity ratio [SMR] 100) and hypertension (SMR 108) were similar to those in the United States of America National Health and Nutrition Examination Survey (NHANES reference SMR 100), while those of diabetes (SMR 83) and metabolic syndrome (SMR 72) were somewhat lower. Anaemia was three times more common than in the USA (SMR 310). Diabetes, hypertension, dyslipidaemia, obesity and the metabolic syndrome were associated with affluence and female sex. Arthritis, anaemia, dementia and stroke were strongly age-associated and these conditions were also the main independent contributors to disability.

**Conclusions:**

The prevalence of many chronic diseases is similar in predominately low socioeconomic status neighbourhoods in the Dominican Republic to that in the USA. Prevalence of age-associated conditions is likely to increase with demographic ageing. There is also scope for increases in cardiovascular disease prevalence, if, as observed in other settings undergoing the epidemiologic transition, the burden of risk factors shifts towards the less affluent. Monitoring future trends in the prevalence and social patterning of chronic diseases may help to assess the effectiveness and equity of primary and secondary prevention strategies. Specific recommendations from our research include identifying and targeting the causes of anaemia among older people, and addressing women's health disadvantages.

## Background

With demographic ageing and the accompanying health transition, chronic diseases are assuming progressively greater significance in countries with low and middle incomes. They are already the leading cause of death in all world regions apart from sub-Saharan Africa. Of the 35 million chronic disease deaths in 2005, 80% occurred in countries with low or middle incomes [1], partly because most of the world's older people live in these regions - 60% now rising to 80% by 2050. However, changing patterns of risk exposure also contribute. Latin America exemplifies the third stage of health transition. As life expectancy improves, and high fat diets, cigarette smoking and sedentary lifestyles become more common, so cardiovascular diseases have maximum public health salience - more so than in stage two regions (China and India) where risk exposure is not yet so elevated, and in stage four regions (Europe) where public health measures have reduced exposure levels [2]. The INTERHEART cross-national case-control study suggests that risk factors for myocardial infarction operate equivalently in all world regions, including Latin America [3].

In a bibliometric analysis of low and middle income country journals covering the period 1998-2003 more than 40% of articles focused on chronic disease research, cardiovascular diseases and cancers being the most popular topics [4]. However, the Latin American region was underrepresented. Chronic disability and its determinants have received comparatively little attention, in research, policy or practice. While cardiovascular diseases and cancers contribute mainly to mortality, much of the burden of other chronic diseases (dementia, mental disorders, diabetes and stroke) arises from years lived with disability [5]. Despite the growing interest in chronic diseases in low and middle income countries [6,7], there is limited information available on their prevalence and impact, and most comprehensive studies focus exclusively or mainly upon young and middle-aged adults [8-10]. Older people contribute 72% of all deaths [5], and 14% of all years lived with disability linked to chronic diseases in low and middle income countries.

Health policy should be informed by precise estimates of disease prevalence and burden. These are lacking for most chronic diseases in most countries with low and middle incomes, particularly for older adults. The 10/66 Dementia Research Group studies in seven Latin American countries, India and China aim to chart the progress of the health transition and its impact upon older persons [11]. This report, from the Dominican Republic, is a comprehensive population-based study of the prevalence and social patterning of chronic disease diagnoses, risk factors and impairments among older people, and their contribution to disability.

## Methods

### Setting

The Dominican Republic shares the Caribbean island of Hispaniola with Haiti. The population is 9.4 million, and 0.5 million (5.7%) are aged 65 and over [12]. Life expectancy is 71 years for men and 75 for women. It is one of the poorest and most unequal of Latin American countries. The per capita GDP (purchasing power parity) is US$ 9,200; 42% of the population live below the poverty line, one third of whom in extreme poverty. Pension coverage, at only 18% of the economically active population, is one of the lowest in Latin America. Community health care is provided by the government through 'primary attention units'. Consultations are free, but medicines must be paid for. Despite low medical insurance coverage, private healthcare is widely patronised.

### Study design and catchment area

A one-phase cross-sectional whole population catchment area survey of all those aged 65 years and over in geographically defined districts in Santo Domingo. Ethical approval for the survey was provided by the research ethics committee for the Institute of Psychiatry, King's College London, and the Bioethics National Committee for Research in the Dominican Republic. Precision calculations indicated that a sample of 2,000 would allow estimation of a dementia prevalence of 2.5% with a precision of ± 0.9%. Catchment area sites were selected purposively, middle-class or high-income areas were avoided. The catchment areas selected were Villa Francisca, San Carlos, San Antón, Mejoramiento Social and Santa Barbara. After defining boundaries, mapping was carried out to identify and locate households. All eligible participants (inclusion criterion age 65 and over) were identified and invited to participate. Age was formally determined on revisit for the interview. Participants were recruited following informed consent or on the basis of a relative's agreement in case of lack of capacity for consent due to dementia. Interviews were carried out in participants' own homes. All participants received the full assessment lasting approximately two to three hours. Ethical approval for the survey was provided by the King's College London research ethics committee, and the Bioethics National Committee for Research in the Dominican Republic.

### Measures

The components of the 10/66 Dementia Research Group baseline population-based survey protocol [11,13] that are relevant to this paper are:

1) a structured clinical mental state interview, the Geriatric Mental State, which applies a computer algorithm (Automated Geriatric Examination for Computer Assisted Taxonomy - AGECAT) [14], identifying organicity (probable dementia), depression, anxiety and psychosis

2) a cognitive test battery comprising the Community Screening Instrument for Dementia (CSI'D') COGSCORE [15] incorporating the Consortium to Establish a Registry for Alzheimer's Disease (CERAD) animal naming verbal fluency task, and the modified CERAD 10 word list learning task with delayed recall [16]

3) The CSI'D' informant interview [15], for evidence of cognitive and functional decline

4) a participant health and risk factor interview covering self-reported diagnoses (including diabetes, stroke, heart attack, angina and hypertension), impairments and disability

5) a fasting blood sample analysed for lipids, glucose and haemoglobin (Sysmex K800 hematology autoanalyser)

6) a physical examination, including systolic and diastolic blood pressure (the mean of two sitting assessments) and anthropometry

Information from these assessments was used to identify describe health states (diagnoses, impairments and risk factors), as follows:

### Diagnoses

a) Dementia according to either the 10/66 dementia diagnosis algorithm [17] or Diagnostic and Statistical Manual of Mental Disorders, 4th. Edition (DSM-IV) dementia criterion [18].

b) Depression - International Classification of Diseases 10^th ^revision (ICD-10) depressive episode (mild, moderate or severe), ascertained using the Geriatric Mental State [14].

c) Self-reported stroke, angina and myocardial infarction (an answer of 'yes' to the questions "have you ever been told by a doctor that you had a stroke/heart attack/angina?")

d) Chronic obstructive pulmonary disease, defined as having a chronic cough, productive of sputum for three or more months

e) Diabetes - either a self-reported diagnosis of diabetes (an answer of 'yes' to the question "have you ever been told by a doctor that you have diabetes?"), and/or a blood glucose of > 7 mmol/l from the survey fasting blood sample

f) Hypertension - either a self-reported diagnosis of hypertension, currently under treatment (an answer of "yes" to the question "have you ever been told that you have raised blood pressure?" and "yes" to the questions "were you started on treatment?" and "are you still on treatment?") and/or meeting World Health Organization/International Society of Hypertension criteria (systolic blood pressure > = 140 mm Hg and/or diastolic blood pressure > = 90 mm Hg on current survey examination.

g) Anaemia - defined as haemoglobin < 12 g/dl for women and 13 g/dl for men

### Physical impairments

Self-reported arthritis or rheumatism; eyesight problems; hearing difficulty or deafness; persistent cough; breathlessness, difficulty breathing or asthma; high blood pressure; heart trouble or angina; stomach or intestine problems; faints or blackouts; paralysis, weakness or loss of one leg or arm; skin disorders (pressure sores, leg ulcers or burns) [19]. Each impairment was rated as present if it interfered with activities 'a little' or 'a lot'.

### Disability

Activity limitation and participation restriction measured by the World Health Organization Disability Assessment Schedule (WHODAS 2.0) [20], developed as a culture-fair assessment tool for use in cross-cultural comparative epidemiological and health services research. Disability days were also ascertained and dichotomised at 15 or more days in the last month, to indicate severe disability.

### Risk factors

a) Metabolic syndrome according to the criteria proposed by the Third Report of the National Cholesterol Education Program: presence of three or more of the following;

1. Central obesity as measured by waist circumference: Men > 40 inches, Women > 35 inches.

2. Fasting triglycerides > = 150 mg/dL.

3. HDL cholesterol: Men < 40 mg/dL, Women < 50 mg/dL (Cholesterol subfractions were not analysed, so we have instead substituted the criterion of total cholesterol > = 5.2 mmol)

4. Blood pressure > = 130/85 mmHg.

5. Fasting glucose > = 110 mg/dL

b) alcohol use (hazardous drinking currently, and before the age of 60),

c) lifetime smoking - 20 or more pack years of lifetime exposure

d) self-reported exercise - taking no walks of 500 metres or more in the past month

### Analyses

1) We report the prevalence of each health state by age and sex using Stata 9.2 survey commands to generate robust standard errors and 95% confidence intervals, taking account of household clustering. We used indirect standardization (by age, or sex, or age and sex as feasible) to compare our prevalence estimates for diabetes, hypertension, metabolic syndrome, stroke and anaemia with those from the United States National Health and Nutrition Examination Survey (NHANES) [21-25], and for dementia with those from the EURODEM consortium European meta-analysis [26], calculating standardized morbidity ratios with 95% confidence intervals

2) We describe the association between age in years and each health state, controlling for sex, and the association with sex controlling for age in years. We also report the association between socioeconomic position indicated by quarters of household assets controlling for age in years and sex. For these analyses we used Poisson regression working models to generate prevalence ratios, adjusted for household clustering.

3) We describe independent associations between diagnoses and impairments and disability using zero inflated binomial regression for the WHODAS total score (correcting for zero inflation and overdispersion) and Poisson regression for the dichotomous outcome of 15 or more disability days in the last month. The resulting prevalence ratios, together with the prevalence of the exposures (diagnoses and impairments were used to calculate population attributable prevalence fractions using the STATA aflogit command, as an index of the salience of each health condition to the prevalence of severe disability at the population level.

## Results

### Sample characteristics

Door-knocking of the five catchment areas yielded 2117 persons eligible for the study; 2011 (95%) provided informed consent and were interviewed. Of these 1483 (74%) provided fasting blood samples. The principal characteristics of the participants are provided in Table 1. Their median age was 74 years (interquartile range 69 to 80 years, total range 65 to 104 years). Two thirds of the participants were female (65.9%). The large majority (71.0%) had not completed primary education. Living alone was unusual (12.6%); most lived in two to four person households. A high proportion of participants were separated or divorced (23.1%), with only 29.1% currently married. Those providing blood samples were more likely to be female (68% vs. 60%), depressed (15% vs. 11%), there were no differences in age, education, household assets, blood pressure levels or dementia diagnoses.

**Table 1:** Sociodemographic and (selected) health characteristics of the sample, and the sub-groups for whom blood samples were, and were not taken

Characteristics (number and % unless otherwise stated)	Whole sample (n = 2011)	Blood sample taken (n = 1451)	Blood sample not taken (n = 560)	Statistical test comparing sub-groups with and without blood samples
Age median (25^th ^and 75^th ^centiles)	74 (69-80)	74 (69-81)	74 (69-80)	Z = -1.46, p = 0.14

Female sex	1324 (65.9%)	316 (60.0%)	1008 (68.0%)	X^2 ^= 11.2, p = 0.001

Education (MV^1 ^= 19)				

None	392 (19.7%)	98 (18.8%)	294 (20.0%)	X^2 ^= 0.8, p = 0.39

Some	1022 (51.3%)	268 (51.5%)	754 (51.2%)	

Completed primary	370 (18.6%)	97 (18.7%)	273 (18.5%)	

Secondary or tertiary	208 (10.5%)	57 (11.0%)	151 (10.3%)	

Living alone	254 (12.6%)	79 (15.0%)	175 (11.8%)	X^2 ^= 3.5, p = 0.06

Marital status (MV = 15)				

Never married	139 (7.0%)	44 (8.4%)	95 (6.5%)	X^2 ^= 4.8, p = 0.19, 3 df

Divorced/separated	465 (23.1%)	133 (25.4%)	332 (22.6%)	

Widowed	806 (40.4%)	198 (37.8%)	608 (41.3%)	

Currently married	586 (29.1%)	149 (28.45)	437 (29.7%)	

Assets				

0-4	648 (32.2)	167 (31.6%)	481 (32.4%)	X^2 ^= 0.3, p = 0.58

5	444 (22.1)	115 (21.8%)	329 (22.2%)	

6	733 (36.4)	194 (36.7%)	539 (36.3%)	

7	186 (9.2%)	52 (9.8%)	134 (9.0%)	

Three or more limiting illnesses (MV = 2)	465 (23.1%)	115 (21.8%)	350 (23.6%)	X^2 ^= 0.7, p = 0.40

Depression	278 (13.8%)	56 (10.6%)	222 (15.0%)	X^2 ^= 6.2, p = 0.01

Dementia	235 (11.7%)	68 (12.9%)	167 (11.3%)	X^2 ^= 1.0, p = 0.32

Systolic blood pressure mean (SD) (MV = 20)	136.2 (20.1)	136.3 (20.6)	136.2 (20.0)	F = 0.56, p = 0.97, 1989

Waist circumference mean (SD)(MV = 25)	92.3 (12.9)	91.6 (13.6)	92.5 (12.6)	F = 0.53, p = 0.18, 1984

1. MV = missing values

### Prevalence of diagnoses and impairments

The most prevalent chronic disease diagnoses were hypertension (73.0%), anaemia (35.0%), diabetes (17.5%), depression (13.8%) and dementia (11.7%). Cardiovascular diseases; stroke (8.7%), myocardial infarction (1.7%) and angina (1.2%) were less common (Table 2). The most prevalent organ and system impairments were eyesight problems (39.6%), arthritis or rheumatism (36.7%), stomach or intestine problems (19.3%) and hearing problems (12.7%). Respiratory problems, heart trouble, limb problems, faints or blackouts and skin disorders all had a prevalence of 10% or less (Table 3). Standardizing for age and sex, the prevalence of anaemia was more than three times higher than that in the United States of America, (Table 4). Dementia and stroke prevalences were similar to those recorded in Europe and the United States of America respectively. The prevalence of diabetes and metabolic syndrome were somewhat lower, and that of hypertension slightly higher than in the United States of America.

**Table 2:** Prevalence of Diagnoses by Age and Sex

		Prevalence of diagnoses by age and sex (% with 95% confidence intervals)				
Diagnoses	Sex	65-69 N = 533	70-74 N = 520	75-79 N = 397	80+ N = 561	All N = 2011
Dementia (10/66 criterion)	F	3.5 1.6, 5.4	7.1 4.3, 9.9	11.7 7.8, 15.5	25.5 21.2, 29.8	12.5 10.8, 14.3
	M	4.8 1.7, 7.8	6.1 2.8, 9.5	14.5 8.5, 20.5	17.2 11.5, 22.9	10.1 7.8, 12.3
Stroke	F	4.7 2.4, 6.9	9.0 5.9, 12.1	5.3 2.6, 7.9	10.7 7.7, 13.8	7.6 6.2, 9.1
MV^1 ^= 6	M	9.0 4.9, 13.1	13.8 8.9, 18.6	7.6 3.1, 12.2	11.9 7.0, 16.8	10.8 8.5, 13.2
Myocardial infarction	F	1.7 0.4, 3.1	2.2 0.6, 3.8	2.3 0, 5, 4.0	1.5 0.3, 2.7	1.9 1.2, 2.6
MV = 35	M	1.6 0.0, 3.4	1.5 0.0, 3.3	1.5 0.0, 3.6	1.2 0.0, 2.8	1.5 0.6, 2.4
Angina	F	1.2 0.0, 2.3	2.2 0.6, 3.8	0.4 0.0, 1.1	1.3 0.2, 2.4	1.3 0.7, 1.9
MV = 25	M	1.1 0.0, 2.5	0.5 0.0, 1.5	1.5 0.0, 3.6	1.8 0.0, 3.8	1.2 0.4, 2.0
Chronic obstructive pulmonary disease	F	6.1 3.6, 8.7	7.1 4.3, 9.9	6.0 3.2, 8.9	5.6 3.3, 7.9	6.2 4.9, 7.5
MV = 3	M	6.4 2.9, 9.9	6.6 3.1, 10.1	9.9 4.8, 15.0	10.1 5.6, 14.7	8.1 6.0, 10.1
Hypertension (meets ISH criteria, and/or currently on treatment)	F	71.0 66.2, 75.8	76.4 71.8, 81.0	75.2 70.0, 80.3	77.6 73.4, 81.8	75.1 72.7, 77.5
MV = 13	M	67.7 61.1, 74.4	72.2 65.9, 78.5	68.2 60.2, 76.3	67.3 60.1, 74.5	69.0 65.5, 72.5
Diabetes (self-reported diagnosis and/or blood glucose of > 7 mmol/l)	F	20.5 15.7, 25.4	22.2 17.0, 27.3	15.3 10.3, 20.2	15.6 11.4, 19.8	18.4 16.0, 20.9
	M	20.5 13.6, 27.3	16.5 10.2, 22.9	14.4 7.2, 21.7	10.1 4.7, 15.5	15.6 12.3, 18.9
Anaemia	F	30.7 25.1, 36.2	25.5 20.0, 31.0	38.8 32.1, 45.6	39.0 33.4, 44.6	33.4 30.5, 36.3
MV = 10	M	28.8 21.1, 36.5	31.8 23.9, 39.8	40.0 29.9, 50.1	54.6 45.7, 63.6	38.3 33.8, 42.7
ICD-10 depression	F	14.0 10.3, 17.7	13.9 10.1, 17.8	16.2 11.7, 20.6	16.8 13.1, 20.5	15.3 13.2, 17.2
	M	8.5 4.5, 12.4	6.6 3.1, 10.1	16.0 9.7, 22.3	15.4 9.9, 20.8	11.1 8.7, 13.4

1. MV = missing values

**Table 3:** Prevalence of Impairments and Disability by Age and Sex

Impairments and disability		Prevalence of impairments by age and sex (% with 95% confidence intervals)				
	Sex	65-69 N = 533	70-74 N = 520	75-79 N = 397	80+ N = 561	All N = 2011
Arthritis or rheumatism	F	42.6 37.3, 47.8	42.7 37.3, 48.1	47.4 41.4, 53.4	44.6 39.8, 49.5	44.2 41.5, 46.9
MV^1 ^= 3	M	20.7 14.9, 26.5	19.9 14.3, 25.5	22.9 15.7, 30.1	25.6 18.9, 32.3	22.1 19.0, 25.2
Eyesight problems	F	38.5 33.3, 43.6	37.2 31.8, 42.5	39.6 33.7, 45.5	49.2 44.3, 54.2	41.6 38.9, 44.2
MV = 3	M	30.7 24.1, 37.3	31.1 24.6, 37.6	36.6 28.4, 44.9	46.4 38.9, 53.9	35.8 32.2, 39.4
Hearing difficulty or deafness	F	8.5 5.5, 11.4	8.7 5.6, 11.7	11.3 7.5, 15.1	19.1 15.3, 23.0	12.2 10.5, 14.0
MV = 3	M	8.5 4.5, 12.4	11.7 7.2, 16.2	11.5 6.0, 17.0	23.2 16.8, 29.6	13.6 11.0, 16.2
Persistent cough	F	8.5 5.5, 11.4	10.8 7.4, 14.2	10.5 6.8, 14.2	12.2 9.0, 15.4	10.6 8.9, 12.2
MV = 3	M	10.6 6.2, 1.5	7.1 3.5, 10.8	9.2 4.2, 14.1	12.6 15.5, 17.6	9.9 7.6, 12.0
Difficulty breathing, breathlessness or asthma	F	8.7 5.8, 11.7	9.3 6.1, 12.4	9.8 6.2, 13.3	12.0 8.8, 15.2	10.0 8.4, 11.7
MV = 2	M	10.1 5.8, 14.3	7.1 3.5, 10.8	6.9 2.5, 11.2	9.5 5.1, 13.9	8.5 6.4, 10.6
Heart trouble or angina	F	5.0 2.7, 7.3	5.9 3.3, 8.5	3.4 1.2, 5.6	4.8 2.7, 7.0	4.8 3.7, 6.0
MV = 3	M	2.6 0.4, 4.9	4.1 1.3, 6.9	6.9 2.5, 11.2	4.2 1.1, 7.2	4.2 2.7, 5.8
Stomach or intestine problems	F	19.5 15.3, 23.7	22.3 17.7, 26.9	21.9 16.9, 26.9	23.0 18.8, 27.2	21.7 19.5, 23.9
MV = 4	M	12.7 7.9, 17.5	16.8 11.6, 22.1	13.7 7.8, 19.6	14.9 9.3, 20.4	14.6 12.0, 17.3
Faints or blackouts	F	3.5 1.6, 5.4	2.8 1.0, 4.6	3.8 1.5, 6.0	5.9 3.6, 8.2	4.1 3.0, 5.2
MV = 5	M	2.6 0.4, 4.9	2.6 0.3, 4.8	1.5 0.0, 3.6	3.0 0.4, 5.6	2.5 1.3, 3.7
Paralysis, weakness or loss of one leg or arm	F	3.2 1.3, 5.1	3.7 1.7, 5.8	1.9 0.2, 3.5	10.1 7.1, 13.0	5.1 3.9, 6.3
MV = 7	M	2.7 0.4, 5.0	6.6 3.1, 10.1	5.3 1.5, 9.2	6.0 2.4, 9.5	5.1 3.5, 6.8
Skin disorders pressure sores, leg ulcers or burns	F	1.7 0.4, 3.1	0.6 0.0, 1.5	1.9 0.2, 3.5	4.3 2.3, 6.4	2.3 1.5, 3.1
MV = 6	M	1.1 0.0, 2.5	0.5 0.0, 1.5	0.8 0.0, 2.3	3.0 0.4, 5.5	1.3 0.5, 2.2
More than 15 disability days in the last month	F	10.5 7.2, 13.7	11.8 8.3, 15.3	14.7 10.4, 18.9	28.5 24.0, 33.0	16.9 14.9, 19.0
MV = 5	M	9.0 4.9, 13.1	13.2 8.5, 18.0	11.5 6.0, 17.0	17.2 11.5, 23.0	12.7 10.2, 15.2

1. MV = missing values (men and women combined)

**Table 4:** Comparison of Prevalence of Health Conditions Between Dominican Republic and Developed Country Settings, With Indirect Standardisation for Age and Sex

Health condition	Criterion	Prevalence (%) in Dominican Republic sample	Source of comparison prevalence data	Standardised for	Standardised morbidity ratio	95% confidence intervals
Diagnosed diabetes	Told by a doctor that he/she has diabetes	14.0%	NHANES 1999-2002, USA [21]	Sex, among those aged 65 and over	88	73, 107
Undiagnosed diabetes	Never told by doctor that he/she has diabetes, and fasting glucose > = 7 mmol/l	3.5%	NHANES 1999-2002, USA [21]	Sex, among those aged 65 and over	65	45, 92
Diabetes	Diagnosed or undiagnosed diabetes	17.5%	NHANES 1999-2002, USA [21]	Sex, among those aged 65 and over	83	70, 97
Hypertension	Blood pressure > = 140/90 or on antihypertensive treatment	73.8%	NHANES 1999-2004, USA [23]	Sex, among those aged 60 and over	108	101, 117
				Age (60-69,70-79, 80+)	105	98, 113
Metabolic syndrome	NCEP - ATP III criteria, but those diagnosed with diabetes considered dysglycaemic and those told they were hypertensive considered hypertensive, regardless of current control	39.6%	NHANES 1999-2002, USA [22]	Age (60-69,70+) and sex	72	64, 80
Stroke	Told by a doctor that he/she has had stroke	8.7%	NHANES 1999-2004, USA [24]	Age (65-74, 75+) and sex	100	81, 123
Dementia	DSM-IV dementia	5.4%	EURODEM meta-analysis, Europe [26]	Age (five year bands) and sex	85	65, 110
Anaemia	WHO criteria - haemoglobin < 12 g/dl in women, < 13 g/dl in men	35.0%	NHANES III, 1988-1994, USA [25]	Age (65-74, 75-84, 85+) and sex	310	262, 373

### The effects of age and sex

Controlling for sex, the prevalence of dementia, stroke, anaemia and depression increased with age, while that of diabetes was lower among older participants (Table 5). Among the impairments, the prevalence of eyesight and hearing problems, cough, limb problems and skin disorders all increased with age, as did overall disability (Table 6). Regarding risk factors, the prevalence of dyslipidaemia, metabolic syndrome and smoking declined with increasing age (Tables 7 and 8). Inactivity increased with age. Controlling for age, stroke and anaemia were more common in men, while hypertension and depression were more common in women (Table 5). Most impairments; arthritis, eyesight problems, stomach or intestine problems, faints or blackouts were more common in women, who were also more likely to report 15 or more disability days (Table 6). Most cardiovascular risk factors; dyslipidaemia, obesity, hyperglycaemia, inactivity, and the metabolic syndrome were also more common among women (Table 8). Smoking, however, was considerably more common among men.

**Table 5:** Effects of Age, Sex and Socioeconomic Position on Diagnoses

	Associations with diagnoses (prevalence ratios with 95% confidence intervals)		
Diagnoses	Age, adjusted for sex	Sex, adjusted for age	Socioeconomic position (quarters of household assets), adjusted for age and sex
Dementia (10/66 criterion)	1.08 1.07, 1.10 p < 0.001	0.89 0.69, 1.15 p = 0.38	0.87 0.77, 0.97 p = 0.02
Stroke MV^1 ^= 6	1.02 1.00, 1.04 p = 0.01	1.45 1.09, 1.93 p = 0.01	1.01 0.88, 1.16 p = 0.88
Myocardial infarction MV = 35	1.00 0.96, 1.03 p = 0.84	0.77 0.37, 1.60 p = 0.49	0.92 0.66, 1.27 p = 0.60
Angina MV = 25	1.02 0.96, 1.07 p = 0.58	0.92 0.39, 2.16 p = 0.86	1.18 0.79, 1.76 p = 0.41
Chronic obstructive pulmonary disease MV = 3	1.01 0.99, 1.03 p = 0.47	1.31 0.93, 1.84 p = 0.12	0.84 0.72, 0.98 p = 0.02
Hypertension (meets ISH criteria, and/or currently on treatment) MV = 13	1.00 1.00, 1.01 p = 0.15	0.92 0.87, 0.98 p = 0.006	1.04 1.01, 1.07 p = 0.004
Diabetes (self-reported diagnosis and/or blood glucose of > 7 mmol/l)	0.98 0.96, 0.99 p = 0.005	0.84 0.66, 1.07 p = 0.15	1.13 1.01, 1.26 p = 0.03
Anaemia MV = 10	1.03 1.02, 1.03 p < 0.001	1.16 1.01, 1.34 p = 0.04	0.85 0.79, 0.91 p < 0.001
ICD-10 depression	1.02 1.01, 1.03 p = 0.003	0.74 0.58, 0.95 p = 0.02	0.70 0.63, 0.78 p = < 0.001

1. MV = missing values

**Table 6:** Effects of Age, Sex and Socioeconomic Position on Impairments and Disability

Impairments and disability	Associations with impairments and disability (prevalence ratios with 95% confidence intervals)		
	Age, adjusted for sex	Sex, adjusted for age	Socioeconomic position (quarters of household assets), adjusted for age and sex
Arthritis or rheumatism MV^1 ^= 3	1.00 1.00, 1.01 p = 0.23	0.50 0.43, 0.59 p < 0.001	0.98 0.92, 1.04 p = 0.47
Eyesight problems MV = 3	1.02 1.01, 1.02 p < 0.001	0.88 0.78, 0.98 p = 0.02	0.90 0.85, 0.95 p < 0.001
Hearing difficulty or deafness MV = 3	1.06 1.04, 1.07 p < 0.001	1.19 0.94, 1.49 p = 0.15	0.91 0.81, 1.02 p = 0.10
Persistent cough MV = 3	1.02 1.00, 1.04 p = 0.02	0.95 0.72, 1.24 p = 0.69	0.86 0.76, 0.98 p = 0.02
Difficulty breathing, breathlessness or asthma MV = 2	1.01 0.99, 1.03 p = 0.26	0.85 0.64, 1.14 p = 0.28	0.75 0.66, 0.86 p < 0.001
Heart trouble or angina MV = 3	1.00 0.98, 1.03 p = 0.98	0.88 0.57, 1.35 p = 0.55	0.81 0.68, 0.97 p = 0.02
Stomach or intestine problems MV = 4	1.01 1.00, 1.02 p = 0.16	0.68 0.55, 0.83 p < 0.001	0.95 0.87, 1.04 p = 0.28
Faints or blackouts MV = 5	1.02 0.99, 1.05 p = 0.14	0.62 0.36, 1.06 p = 0.08	0.73 0.57, 0.93 p = 0.01
Paralysis, weakness or loss of one leg or arm MV = 7	1.06 1.03, 1.08 p < 0.001	1.07 0.72, 1.59 p = 0.72	0.86 0.71, 1.03 p = 0.10
Skin disorders pressure sores, leg ulcers or burns MV = 6	1.06 1.02, 1.10 p = 0.001	0.62 0.30, 1.31 p = 0.21	0.58 0.42, 0.81 p = 0.001
More than 15 disability days in the last month MV = 5	1.05 1.04, 1.06 p < 0.001	0.80 0.63, 1.00 p = 0.05	0.89 0.80, 0.99 p = 0.03

1. MV = missing values

**Table 7:** Prevalence of Risk Factors by Age and Sex

		Prevalence of risk factors by age and sex (% with 95% confidence intervals)				
Risk factors	Sex	65-69 N = 400^1^ N = 533^2^	70-74 N = 382^1^ N = 520^2^	75-79 N = 293^1^ N = 397^2^	80+ N = 408^1^ N = 561^2^	All N = 1483^1^ N = 2011^2^
Metabolic syndrome (NCEP criteria)	F	38.2 32.3, 44.1	46.3 40.1, 52.6	37.3 30.6, 44.0	35.7 30.1, 41.2	39.3 36.3, 42.4
	
MV^3 ^= 30	M	23.3 16.0, 30.6	26.2 18.6, 33.7	15.3 7.6, 23.0	15.5 8.9, 22.1	20.7 16.9, 24.4

Metabolic syndrome components						

Triglyceride > 150 mg/dl	F	24.5 19.3, 29.7	24.5 19.1, 29.9	14.4 9.5, 19.3	19.4 14.8, 24.0	21.0 18.5, 23.6
MV = 31	M	19.4 12.5, 26.2	16.2 9.8, 22.5	18.8 10.5, 27.1	10.3 4.8, 15.9	16.1 12.7, 19.4
Total cholesterol > = 5.2 mmol	F	49.4 43.4, 55.5	48.2 41.9, 54.5	47.8 40.8, 54.7	41.0 35.3, 46.7	46.3 43.2, 49.5
MV = 31	M	35.7 27.4, 43.9	36.9 28.6, 45.2	28.2 18.7, 37.8	22.4 14.8, 30.0	31.3 27.0, 35.6
Waist circumference; Men > 40 inches, Women > 35 inches	F	62.8 57.0, 68.7	64.1 58.0, 70.2	58.2 51.3, 65.0	56.5 50.8, 62.3	60.4 57.4, 63.4
MV = 25	M	19.4 12.5, 26.2	20.0 13.1, 26.9	18.8 10.5, 27.1	23.3 15.6, 31.0	20.4 16.7, 24.1
Blood pressure > = 130/85 mmHg	F	59.2 53.2, 65.1	69.1 63.3, 74.9	70.1 63.8, 76.5	68.4 63.0, 73.9	66.4 63.5, 69.4
MV = 20	M	71.9 64.1, 79.7	77.7 70.5, 84.9	64.7 54.5, 74.9	67.2 58.7, 75.8	71.0 66.9, 75.2
Fasting glucose > 110 mg/dl	F	26.0 20.6, 31.3	28.5 22.8, 34.1	30.3 24.0, 36.7	27.0 21.7, 32.1	27.7 24.9, 30.5
MV = 32	M	28.1 20.3, 35.9	27.7 20.0, 35.4	27.1 17.6, 36.5	18.1 11.1, 25.1	25.3 21.3, 29.2

Other risk factors						

Limited exercise (no walks of > 0.5 km in last month)	F	30.1 25.2, 35.0	36.8 31.5, 42.1	35.6 29.8, 41.4	64.9 60.1, 69.7	43.1 40.4, 45.8
MV = 12	M	17.6 12.1, 23.0	24.5 18.5, 30.5	27.3 19.6, 35.1	35.9 28.7, 43.2	25.9 22.6, 29.2
Smoking (> 20 pack years)	F	11.0 7.6, 14.4	10.3 6.8, 13.8	11.8 7.8, 15.7	8.3 5.5, 11.1	10.2 8.5, 11.9
MV = 114	M	35.5 28.6, 42.4	33.0 26.3, 39.7	29.2 21.0, 37.3	28.9 21.9, 35.9	32.0 28.4, 35.6

1. Sample size with blood tests

2. Total sample size

3. MV = missing values (men and women combined)

**Table 8:** Effects of Age, Sex and Socioeconomic Position on Risk Factors

	Associations with risk factors (prevalence ratios with 95% confidence intervals)		
Risk factors	Age, adjusted for sex	Sex, adjusted for age	Socioeconomic position (quarters of household assets), adjusted for age and sex
Metabolic syndrome (NCEP criteria) MV^1 ^= 30	0.93 0.88, 1.00 P = 0.04	0.52 0.43, 0.63 P < 0.001	1.17 1.09, 1.25 P < 0.001

Metabolic syndrome components			

Triglyceride > 150 mg/dl MV = 31	0.88 0.81, 0.96 P = 0.009	0.76 0.60, 0.96 P = 0.02	1.22 1.10, 1.35 P < 0.001
Total cholesterol > = 5.2 mmol MV = 31	0.93 0.88, 0.97 P = 0.002	0.67 0.58, 0.78 P < 0.001	1.09 1.02, 1.16 P = 0.007
Waist circumference; Men > 40 inches, Women > 35 inches MV = 25	0.97 0.93, 1.01 P = 0.14	0.33 0.27, 0.40 P < 0.001	1.11 1.06, 1.17 P < 0.001
Blood pressure > = 130/85 mmHg MV = 20	1.01 0.98, 1.05 P = 0.35	1.07 0.99, 1.15 P = 0.07	1.01 0.97, 1.04 P = 0.74
Fasting glucose > 110 mg/dl MV = 32	0.97 0.90, 1.04 P = 0.49	0.91 0.76, 1.09 P = 0.31	1.14 1.04, 1.24 P = 0.03

Other risk factors			

Limited exercise (no walks of > 0.5 km in last month) MV = 12	1.16 1.13, 1.20 P < 0.001	0.78 0.73, 0.83 P < 0.001	1.00 0.97, 1.03 P = 0.96
Smoking (> 20 pack years) MV = 114	0.93 0.86, 1.01 P = 0.08	3.10 2.54, 3.78 P < 0.001	0.94 0.86, 1.03 P = 0.20

1. MV = missing values

### The effect of socioeconomic position

Controlling for age and sex, dementia, chronic obstructive pulmonary disease, anaemia and depression were each less prevalent with increasing household assets (Table 5). The association was in the opposite direction for diabetes, hypertension, dyslipidaemia, obesity, hyperglycaemia and the metabolic syndrome (Tables 5 and 8). There was a trend for all physical impairments to be inversely associated with assets, statistically significant for eyesight problems, cough, breathing difficulties, faints or blackouts and skin disorders (Table 6). Disability was also strongly inversely associated with assets. We also tested for associations with level of education (detailed results available on request). None of the health conditions positively associated with assets (diabetes, hypertension, dyslipidaemia, obesity, hyperglycaemia and the metabolic syndrome) was associated with higher levels of education. However, most of the conditions negatively associated with household assets were also negatively associated with level of education (dementia, anaemia and depression). There were only non-significant trends towards negative associations between educational level and individual impairments, but there was a significant inverse association with disability.

### Associations with disability

In order of level of contribution, defined by the population attributable prevalence fraction, the strongest independent influences upon disability were arthritis, anaemia, limb impairments, dementia, depression, intestinal problems and stroke (Table 9). None of the indicators of ischaemic heart disease or respiratory impairment was significantly associated with disability.

**Table 9:** Independent Associations Between Diagnoses and Impairments, and Disability

Outcome		WHODAS II disability score			15 or more disability days			
		Median score in the exposed group (interquartile range)	Independent associations between diagnoses and impairments and WHODAS II score ^1^		Proportion (%) with 15 or more disability days- in the exposed group	Independent associations between diagnoses and impairments and 15 or more disability days ^2^		
Exposure	Number and proportion (%) exposed		RR	95% confidence intervals		PR	95% confidence intervals	Population attributable prevalence fraction
**Diagnoses**								
Dementia (10/66 criterion)	235 (11.7%)	27.8 (8.3-58.3)	1.57	1.40, 1.77	79/235 (33.6%)	1.50	1.16, 1.95	10.5%
Stroke	175 (8.7%)	27.8 (8.3-57.6)	1.37	1.21, 1.55	65/175 (37.1%)	1.46	1.12, 1.91	6.5%
Myocardial infarction	35 (1.7%)	19.4 (5.6-38.9)	0.90	0.74, 1.09	7/35 (20.0%)	0.82	0.42, 1.61	
Angina	25 (1.2%)	22.2 (1.4-44.4)	1.23	0.88, 1.73	8/25 (32.0%)	1.50	0.87, 2.57	0.8%
Hypertension	1516 (75.4%)	8.3 (0.0-27.8)	1.00	0.90, 1.11	245/1515 (16.2%)	1.06	0.81, 1.38	5.7%
Chronic obstructive pulmonary disease	137 (6.8%)	22.2 (5.6-41.7)	1.02	0.90, 1.16	36/137 (26.3%)	1.18	0.85, 1.64	1.6%
Diabetes ^3^	260 (17.5%)	13.9 (0.0-30.6)	1.07	0.96, 1.19	48/260 (18.5%)	0.92	0.68, 1.26	Inverse association
Anaemia ^3^	515 (35.0%)	13.9 (0.0-35.4)	1.16	1.06, 1.27	20/84 (23.8%)	1.53	1.16, 2.02	17.1%
ICD-10 depression	278 (13.8%)	33.3 (16.7-50.0)	1.43	1.30, 1.57	91/278 (32.7%)	1.48	1.16, 1.90	9.4%
								
**Impairments**								
Arthritis or rheumatism	737 (36.7%)	19.4 (5.6-33.3)	1.34	1.23, 1.46	167/736 (22.7%)	1.68	1.35, 2.10	22.5%
Eyesight problems	796 (39.6%)	19.4 (5.6-36.1)	1.13	1.04, 1.23	153/795 (19.2%)	1.01	0.81, 1.27	1.8%
Hearing difficulty or deafness	255 (12.7%)	25.0 (5.6-43.7)	1.17	1.05, 1.30	60/255 (23.5%)	1.10	0.80, 1.72	0.9%
Persistent cough	207 (10.3%)	19.4 (5.6-41.7)	1.01	0.90, 1.13	55/206 (26.7%)	1.11	0.83, 1.47	2.0%
Difficulty breathing, breathlessness or asthma	191 (9.5%)	30.6 (11.1-47.2)	1.12	1.00, 1.27	60/190 (31.6%)	1.19	0.88, 1.60	2.8%
Heart trouble or angina	93 (4.6%)	27.8 (11.1-40.9)	1.11	0.96, 1.30	26/93 (28.0%)	1.17	0.79, 1.71	0.9%
Stomach or intestine problems	388 (19.3%)	25.0 (8.3-44.4)	1.27	1.16, 1.39	103/388 (26.5%)	1.33	1.04, 1.71	8.9%
Faints or blackouts	71 (3.5%)	33.3 (18.1-56.3)	1.30	1.10,1.53	29/71 (40.8%)	1.25	0.82, 1.92	1.5%
Paralysis, weakness or loss of one leg or arm	102 (5.1%)	50.0 (27.8-69.4)	1.88	1.67,2.12	60/102 (58.8%)	2.50	1.92, 3.25	11.8%
Skin disorders (pressure sores, leg ulcers or burns)	39 (1.9%)	50.0 (27.8-69.4)	1.70	1.40,2.08	21/39 (53.8%)	2.43	1.57, 3.77	4.3%

1 Zero-inflated negative binomial regression, adjusted for all other diagnoses and impairments other than diabetes and anaemia. N = 1975 (see also footnote 3

2 Poisson regression

3 Parameters for diabetes and anaemia were obtained from a separate model restricted to those participants who had provided a blood sample

## Discussion

In the current study we were able to ascertain the prevalence and distribution of a wide range of chronic disease diagnoses, impairments and underlying risk factors in a large population-based sample drawn from urban catchment areas in Santo Domingo, the capital city of the Dominican Republic. There have been very few previous reports of the prevalence of chronic diseases and their risk factors among older people, in countries with low and middle incomes in Latin America or other regions. In our study, the catchment areas were selected as typical examples of the lower income areas that account for much of Santo Domingo's population. However, findings from this survey cannot be generalised safely to other parts of the city or to the country as a whole. Not all diagnoses were ascertained with equivalent rigour; self-reported clinician diagnoses, for example stroke and heart disease, may have been under-reported with respect to other conditions such as dementia and depression that were identified through clinical interview, and hypertension and diabetes that were identified through blood pressure and fasting glucose measurement respectively. The overall proportion responding, 95%, was very high. However, only 74% of those agreeing to the survey also supplied fasting blood samples that were used to identify diabetes, hyperlipidaemia, metabolic syndrome and anaemia. While most sociodemographic and health variables were not associated with provision of a blood sample, women, depressed participants, and those with three or more limiting impairments were slightly over-represented among those giving blood samples suggesting some potential for non-response bias in the estimation of the prevalence and correlates of these metabolic and haematological disorders.

In the CARMELA study of cardiovascular risk in those aged 25-64 years in seven Latin American cities [8], the prevalences of hypertension, diabetes and metabolic syndrome were similar to developed countries in Venezuela, Argentina and Chile with a lower prevalence in the less developed Latin American countries. The Dominican Republic is just 90 minutes flying time from Miami but purchasing power parity per capita gross domestic product is only one fifth that of the United States of America ($9,200 versus $46,000). Demographic ageing is much less advanced in the Dominican Republic, with just 5.7% of its population aged 65 and over, compared with 12.6% in the United States of America. Nevertheless, the age and sex adjusted prevalence of dementia and stroke seem already to have reached levels seen in Europe and the United States of America. Hypertension is, if anything, slightly more prevalent in the Dominican Republic. The high prevalence of hypertension and stroke may be explained in part by the high levels of African racial admixture seen in many residents of our catchment areas. Only diabetes and the metabolic syndrome were marginally less prevalent than in the United States of America. At the same time, anaemia, a condition strongly linked to dietary deficiency and poverty is more than three times more common in the Dominican Republic. This pattern of morbidity illustrates the 'double burden' of disease in countries undergoing the health transition - as chronic diseases become more prevalent, infectious and nutritional disorders recede but continue to make important contributions to mortality and disability.

Predicting the impact of the demographic and health transitions requires an understanding of the influences of age, sex and socioeconomic status on disease frequency and underlying risk factor exposures. In the Dominican Republic the negative relationship with increasing age for all cardiovascular risk factors other than hypertension suggests either a cohort effect with increasing exposure levels to be anticipated in future aged cohorts, or selective mortality, in which case improved secondary prevention may lead over time to more chronic morbidity and disability. Most of the disorders that were positively associated with older age (arthritis, anaemia, dementia, stroke, limb impairments) also made strong independent contributions to disability. Thus, in this setting, the numbers of frail and dependent older persons are likely to increase rapidly with demographic ageing. We have previously shown that the age-specific prevalence of dependence in the Dominican Republic is already similar to that in developed nations [27]. Unfortunately, social protection for older people in the Dominican Republic is very poorly developed. Pension coverage has been among the poorest in the world. Our survey indicated a particularly high proportion of older people without children available to care [28], both because of infertility and outmigration.

In the United States of America, the age-adjusted prevalence of hypertension [29], diabetes [21] and the metabolic syndrome [22] is similar in men and women. However, women with diabetes and hypertension may be at greater risk of cardiovascular disease [30]. The prevalence of metabolic syndrome in the Dominican Republic was double in older women that of men, mainly due to a marked excess of obesity and dyslipidaemia among women, and a trend towards a higher prevalence of diabetes. This is consistent with findings among younger Latin Americans [8], but contrasts with the higher prevalence among men in many developed country studies, albeit that recent increases in prevalence have been more rapid among younger women [30]. In our study, older women were also more likely to have been diagnosed as hypertensive, but were generally better controlled. Despite the generally increased cardiovascular risk exposure among women, the prevalence of stroke was markedly higher among men in all age groups and there was little effect of sex on ischemic heart disease. The very low levels of smoking among women may have compensated.

Our most striking findings relate to health inequalities. In countries with high incomes, cardiovascular risk factors and diseases are typically associated with poverty. Our assessment of the effects of the effects of socioeconomic position on chronic disease outcomes may have been limited to some extent by the constrained variance in the predominately low social class catchment areas. Nevertheless, among older people in the Dominican Republic this pattern of association was observed, but only for the more age-related and disabling conditions (dementia, anaemia, depression and most of the chronic limiting impairments). Conversely, cardiovascular risk factors (hypertension, diabetes, dyslipidaemia, obesity and the metabolic syndrome) were each associated with relative affluence, and there was no socioeconomic gradient for ischaemic heart disease or stroke. These findings are consistent with associations observed in a nationally representative survey of Thais aged 50 years and older, where poor self-rated health and functional limitation were associated with lower levels of education, income and wealth, but self-reported diagnoses of hypertension and heart disease were over-represented in the more affluent [31]. Urbanization may have important influences on the socioeconomic patterning of health; in the nationally representative Mexican Health and Ageing Study activity limitation was consistently associated with socioeconomic disadvantage in urban but not in rural areas [32]. In Buenos Aires, in a survey of adults of all ages, hypertension diagnosis was associated with low education and income in both sexes, as were obesity and high body mass index in women but not men [33]. In high income countries metabolic syndrome tends to be inversely associated with socioeconomic position in middle age; few studies have included older persons, but in the United States of America NHANES the effect of socioeconomic position was limited to women aged 25-64 [34]. Little research has been conducted in countries with low and middle incomes, but our findings are consistent with reports from India of a higher prevalence of metabolic syndrome among better educated adolescents [35]. With regard to lifestyle risk factors, much more research has been conducted into associations between socioeconomic position and obesity, documenting the progress of the health transition in countries with low and middle incomes - obesity is no longer confined to the more affluent and inverse associations with socioeconomic position are increasingly observed, particularly for women living in countries with per capita GDP greater than $2500 [36]. In Brazil, for example, there was a clear shift in the burden of obesity towards the poor between 1975 and 2003; the prevalence in women remained stable overall, but increased by 26% in the poorest two-fifths and decreased by 10% among the richest three-fifths [37]. The health transition is therefore likely to accentuate health inequalities. In the United States of America, reductions over the last 30 years in hypertension and dyslipidaemia have not lessened socioeconomic differences, and those for smoking and diabetes have increased due to less smoking in high income groups and increases in diabetes prevalence among those with low incomes [38]. The effect of the health transition on health inequalities may be particularly marked among older people. In the longitudinal American's Changing Lives Study [39], socioeconomic differences in health are modest in youth, increase markedly across middle age into young old age, and decline thereafter, a pattern observed consistently in developed country studies. There is evidence from the American's Changing Lives study that compression of morbidity (the increasing tendency for the preservation of health and functioning into late old age) is mainly observed in the most educated, and that this effect is becoming more pronounced over time.

## Conclusions

The prevalence of many chronic diseases and chronic disease risk factors is already nearly as high in low socioeconomic status neighbourhoods in Santo Domingo, Dominican Republic as in the USA. Cautious extrapolation from our cross-sectional data suggests that the overall prevalence of conditions that are strongly age-associated (dementia, stroke and arthritis) is likely to rise in coming years with continued demographic ageing. There is also scope for increases in the prevalence of those cardiovascular risk factors currently associated with relative affluence (diabetes, hypertension, dyslipidaemia, obesity and the metabolic syndrome), if, as has been observed in other settings undergoing the epidemiologic transition, these exposures begin to be concentrated instead among the more numerous economically disadvantaged sectors of the population. This scenario would threaten both an increase in the overall incidence and prevalence of cardiovascular diseases, and in the extent of health inequalities. It will be important to monitor the actual extent and direction of such trends with further epidemiological research, in particular to assess the effectiveness and equity of attempts to improve the prevention and control of chronic diseases. The generally poor health of older women in the Dominican Republic should be a matter of public health concern, and a focus for prevention activities. Controlling for age, women were likelier than men to be hypertensive, dyslipidaemic, obese and underactive and had a higher prevalence of the metabolic syndrome, depression, physical impairments and severe disability. More research into the prevalence and causes of anaemia in older people is indicated, and the government should consider evaluating screening and treatment programmes. Finally, more attention should be given to the social protection of older people, including income security and provision and financing of long term care [40].

## Abbreviations

AGECAT: Automated Geriatric Examination for Computer Assisted Taxonomy; CSI'D': Community Screening Instrument for Dementia; CERAD: Consortium to Establish a Registry for Alzheimer's Disease; DSM-IV: Diagnostic and Statistical Manual of Mental Disorders, 4th. Edition; ICD-10 International Classification of Diseases 10^th ^revision; WHODAS 2.0: World Health Organization Disability Assessment Schedule; NHANES: National Health and Nutrition Examination Survey

## Competing interests

The 10/66 Dementia Research Group works closely with Alzheimer's Disease International (ADI), the non-profit federation of 77 Alzheimer associations around the world. ADI is committed to strengthening Alzheimer associations worldwide, raising awareness regarding dementia and Alzheimer's Disease and advocating for more and better services for people with dementia and their caregivers. ADI is supported in part by grants from GlaxoSmithKline, Novartis, Lundbeck, Pfizer and Eisai. Daisy Acosta is currently the Chair of ADI.

## Authors' contributions

All of the authors worked collectively to develop the protocols and methods described in this paper. MJP led the research group and CPF acted as research coordinator. DA was the principal investigator responsible for the field work in the Dominican Republic. MJP wrote the first draft with the assistance of DA and RR. These three authors also conducted the analyses. Other authors reviewed the manuscript, provided further contributions and suggestions. All authors read and approved the final manuscript.

## Pre-publication history

The pre-publication history for this paper can be accessed here:

http://www.biomedcentral.com/1471-2458/10/344/prepub
